# Acute Effects of Whole-Body Electromyostimulation during a Single Maximal Strength Training Session

**DOI:** 10.3390/ijerph192113753

**Published:** 2022-10-22

**Authors:** Valentín E. Fernández-Elías, David Tobía, Anel Recarey, Álvaro Fernández, Vicente J. Clemente-Suárez, Silvia Burgos-Postigo

**Affiliations:** 1 Faculty of Sport Sciences, Universidad Europea de Madrid, 28670 Villaviciosa de Odón, Spain; 2 Grupo de Investigación en Cultura, Educación y Sociedad, Universidad de la Costa, Barranquilla 080007, Colombia

**Keywords:** WB-EMS, creatine kinase, acute effects, muscle damage, heart rate variability

## Abstract

Whole-body electromyostimulation (WB-EMS) training is effective in improving training adaptation. However, WB-EMS may have side effects and contraindications that can lead to excessive muscle damage and physiological impairment. This randomized crossover study aimed to analyze the acute effects of WB-EMS on muscle damage, autonomic modulation and performance during a single maximal strength session in physically active participants. Twenty healthy and physically active participants randomly performed three maximal strength training sessions (90% 1RM) consisting of bench presses and squat exercises, with a continuous stimulus, a coordinated stimulus with concentric and eccentric phases, and without WB-EMS. Data showed no significant differences between the trials for muscle damage (blood creatine kinase levels), lactate blood levels and performance after exercise. Likewise, the heart rate, blood oxygen saturation and the rate of perceived exertion were similar during exercise between trials. The heart rate variability analysis also showed a similar autonomic response among the trials. Training with WB-EMS seemed to be safe at the observed time intervals while offering a stimulus similar to regular training in physically active participants, regardless of the delivery of the electrical stimuli. More studies are needed to assess the effectiveness of WB-EMS in improving exercise adaptations during training programs.

## 1. Introduction

Electromyostimulation (EMS) training can be an effective and alternative method to traditional strength training, to improve muscle strength, speed strength, sprinting, jumping and athletic performance [1,2,3,4,5]. However, this technology can be applied only to specific muscles with single electrodes, and usually in a passive way. Technology improvements lead to a new generation of devices (whole-body electromyostimulation (WB-EMS)) that allow to exercise the whole body, or previously selected muscles or body areas at the same time, and simultaneously. This WB-EMS equipment has been recently developed as an alternative form of physical exercise, to improve fitness and health, despite the limited scientific evidence about its security and efficacy [6]. Nevertheless, there is substantial research focusing on local EMS, while WB-EMS research is scarce [6], with most research studying its effects on a special population (i.e., sedentary, aged or ill) and little research analyzing the performance effects on trained, physically active people [7].

Despite the extensive evidence showing the benefits of EMS on muscle performance, WB-EMS training was catalogued as unsuitable and dangerous for users’ health [8], due to three case studies reporting users went to the medical service describing intense pain and blood in their urine, after a WB-EMS training session [8,9,10]. They were diagnosed with rhabdomyolysis, according to their extremely high (i.e., >10,000 U/L) creatine kinase (CK) blood levels [11]. It is known that the CK blood level is a parameter that indicates muscle damage, it is usually used in training and sport, to control the exercise intensity load and recovery [12], with a breakpoint at 300–500 U/L of the CK release after exercise and recovering its basal level after 24–48 h [13].

Despite the safety concerns of WB-EMS training, it has been proposed as an alternative method to maintain muscle mass in older adults with osteopenia and sarcopenia [14,15]. Moreover, other studies have shown health improvements after analyzing the effects of WB-EMS on the energy expenditure [16] and the body composition [17] of a special population, suggesting that the CK blood levels increase after a WB-EMS session but not dangerously. Therefore, criticism regarding the safety of WB-EMS training might be based on case-reported studies where possibly non-professional use of WB-EMS was applied. In the physically active population, an increase in the CK blood levels was found after a football training session [7] and stimulated the contraction of the quadriceps [18] when using WB-EMS, compared to voluntary exercise. Nevertheless, the CK levels were under the damage threshold of 5000 U/L [19]. Other authors found significant differences in the isokinetic muscle function and balance functions in WB-EMS training [20], but there is still controversy about whether WB-EMS is a hazard and should not be used as a training modality.

The misuse of the WB-EMS devices by inexperienced personnel or their use in non-advisable people can be observed in the aforementioned case studies and experimental studies that showed an excessive increase in the CK levels. WB-EMS training must be individually regulated and adapted to each user, since it is necessary to understand that this method involves an increased training load [14,21,22]. Thus, it should be carried out in physically active people who can assimilate that increase in the training load safely. Further, one of the possible reasons for the excessive muscle damage is the increased tension time of the muscle fibers by being in constant contraction because the WB-EMS has been typically set as a continuous stimulus, not respecting the concentric and eccentric phases of the movement.

Therefore, the aim of this study was to investigate the effects of the continuous stimulus WB-EMS (Cont WB-EMS), the coordinated stimulus WB-EMS with concentric and eccentric phases (Coord WB-EMS) and the absence of WB-EMS (Control) on muscle damage, fatigue and performance, during a single maximal strength session in the physically active population. We hypothesized that the WB-EMS trials would lead to a similar physiological impairment compared to the Control trial.

## 2. Materials and Methods

### 2.1. Participants

Twenty (ten men and ten women) healthy and physically active participants volunteered to join in this randomized crossover study. Women were regularly menstruating and not using oral contraceptives. All participants had previous experience in resistant training of 3–5 days per week for at least a year before the beginning of the study. The performance and biological variables were not different between males and females at any time point during the experiment, and thus, the results were pooled, analyzed and reported with all participants grouped. The subjects were fully informed about the experimental procedures and the possible risks and discomforts associated with the experiment before they gave their written informed consent to participate. 

### 2.2. Experimental Design

The study procedure was carried out at the Training Lab of the University. All participants underwent three experimental trials in a random counterbalanced order, separated by three days between them. Trials were a single maximal strength training session (90% 1RM), consisting of two exercises, bench presses (BPs) for the upper body and squats (SQs) for the lower body, both performed in the Smith machine. The training intensity of 90% 1RM was chosen, in order to elicit high-intensity load neuromuscular demands. Each exercise consisted of five sets of five repetitions and 3 min of rest between sets. Each trial was performed with a different protocol of WB-EMS: continuous stimulus (85 Hz, 250/350 µs; Cont WB-EMS); coordinated stimulus with concentric and eccentric phases (85 Hz, 250/350 µs, 1-s impulse and 2-s rest; Coord WB-EMS). These times were previously determined (pilot test) to coincide with the concentric and eccentric phases for the velocity of the displacement at a 90% 1RM load; and one session without WB-EMS (Control). In the WB-EMS trials the device was set off during the 3 min recovery periods between exercise sets. 

### 2.3. WB-EMS Familiarization and Determination of 1RM

Prior to the beginning of the experimental trials, all participants underwent one training familiarization session with the WB-EMS equipment (MyoFX EMS System, Madrid, Spain), in order to avoid a bias of learning and possible discomforts, caused by the electromyostimulation. The next day, the subjects performed a test to determine the individual 1RM. Based on this information, the load corresponding to the 90% of the 1RM was set for the experimental trials. This test was performed through the measurement of the mean propulsive velocity associated with four submaximal loads for the BPs and the SQs, according to the protocol previously described by Pallares et al. [23]. Briefly, the participants were instructed to perform the eccentric phase of both exercises in a slow and controlled manner (0.45–0.65 m/s), remain paused for 2 s at the bar holders momentarily releasing the weight of the load, and thereafter to perform a purely concentric action pushing back up at the maximal intended velocity. The momentary pause imposed between the eccentric and concentric actions was designed to minimize the contribution of the stretch-shortening cycle (i.e., rebound effect) and allow for more reliable and consistent measurements. To measure the mean propulsive velocity, we used an inertial measurement unit (FreeSense, Sensorize Ltd., Rome, Italy) containing a 3D accelerometer and a 3D gyroscope (±6 g and ±500 deg·s^−1^ of full range, respectively; 100 samples·s^−1^), placed in the bar, that was previously validated [24].

### 2.4. Experimental Protocol

Two days before the onset of each experimental trial, the participants refrained from exercising, alcohol and caffeine use [25]. Each participant started the three experimental trials at the same time of day to avoid any circadian rhythm interaction [26]. Prior to the exercise, the participants lay down on a stretcher for 10 min to monitor the heart rate variability (HRV), according to previous studies [27]. Then, the basal CK and lactate levels were measured through the capillary blood. Then, the participants realized a standardized warm-up consisting of 10 min of low intensity cycling and three submaximal load sets of BPs and SQs. Following the warm-up, the participants performed a series of physical tests: squat jumps, countermovement jumps, Abalakov jumps, isometric handgrip strength and a punching speed test. Subsequently, the participants began the exercise experimental trial (Cont WB-EMS, Coord WB-EMS or the Control trial). The heart rate (HR), blood oxygen saturation (BOS) and rating of the perceived exertion (RPE) were monitored at the end of each set. At the end of the experimental trial, the participants performed the physical test, then the CK and lactate levels were measured and finally the HRV was again monitored. after the next day, the CK blood level was again measured 24 h after the end of the experimental trial to analyze the muscle mass degradation. 

### 2.5. Measurements

The heart rate variability (HRV) was measured. The participants lay quietly on a stretcher with a stable breathing rate for 10 min wearing a heart rate monitor with the R-R measurement function (Ambit Peak 3, Suunto, Vantaa, Finland), that was previously validated [28]. The HRV data were later analyzed using Kubios HRV software (v3.0.2, University of Kuopio, Kuopio, Finland). The parameters analyzed were:

Time Domain Variables

RMSSD: The root mean square of the successive differences of the R-R intervals in milliseconds.

PNN50: The proportion (%) of NN50, divided by the total number of the Normal-to-Normal bit (R-R) intervals.

Frequency Domain Variables

LF: The low-frequency band in normalized units.

HF: The high-frequency band in normalized units.

LF/HF ratio: A ratio of the Low Frequency to High Frequency.

Nonlinear Variables

SD1: Short-term variability in milliseconds.

SD2: Long-term variability in milliseconds.

ApEN: Approximate entropy that measures the complexity or irregularity of the signal.

SampEn: Sample entropy is a modification of ApEn, used for assessing the complexity of the physiological time-series signals, diagnosing diseased states.

The creatine kinase (CK) and the lactate capillary blood levels were analyzed before and after the experimental trials. CK was also measured 24 h after the end of the experimental trials. The capillary blood from the finger was obtained to assess the CK (35 µL) and lactate (5 µL) blood levels. CK was analyzed using an automatic reflection photometer (Reflotron Plus, Roche, Munich, Germany). Lactate was evaluated using a lactate analyzer (Lactate Plus, Nova Biomedical, Zürich, Switzerland).

The heart rate (HR) and blood oxygen saturation (BOS) were monitored after each exercise set, using a pulse oximeter (MD300C15D, Prim, Reus, Spain). The use of a heart rate monitor, during the exercise was discarded because of the interference with the WB-EMS system. 

The perceived exertion (RPE) using the modified 10-point Borg scale, was obtained during the exercise and after each exercise set was analyzed [29].

The squat Jump (SJ), countermovement jump (CMJ) and Abalakov jump (ABK) tests were performed before and after the experimental trials. The highest jump out of three attempts was determined using an inertial measurement unit (FreeSense, Sensorize Ltd., Rome, Italy) attached to the participants’ waists using a specific belt [24].

The isometric handgrip strength (IHS) was measured using a calibrated handgrip dynamometer (Takei 5101, Tokyo, Japan). Participants sat with 0 degrees of shoulder flexion, 0 degrees of elbow flexion, and the forearm and hand in a neutral position. The highest value out of two attempts was recorded as the maximum voluntary handgrip strength.

Punching speed (PS) was defined as the number of punches hit for 5 s. To measure this variable, the participants were recorded while hitting a punching bag for 5 s using the camera of an electronic tablet (iPad Air 2, Apple, Cupertino, CA, USA). Then, the videos recorded were analyzed through slow-motion video reproduction by two different researchers to determine the number of punches performed. Punches with a wrong technique (punches not hitting the bag or not recovered to the initial position) were discarded. 

### 2.6. Statistical Analysis

Data are presented as mean ± SD. The Kolmogorov–Smirnov test was used to confirm a normal distribution of data. A two-way ANOVA (experimental trial × time) was run to analyze the differences in all of the reported variables. Following a significant F test (Greenhouse–Geisser adjustment for sphericity), the differences between the means were identified using the Bonferroni correction for the type-I error when the trial × time interaction was significant. The statistical significance was set at *p* < 0.05. The data analysis was performed using SPSS Statistics (v21.0, IBM, Armonk, NY, USA).

## 3. Results

### 3.1. Creatine Kinase and Lactate Blood Levels

**Figure 1:**
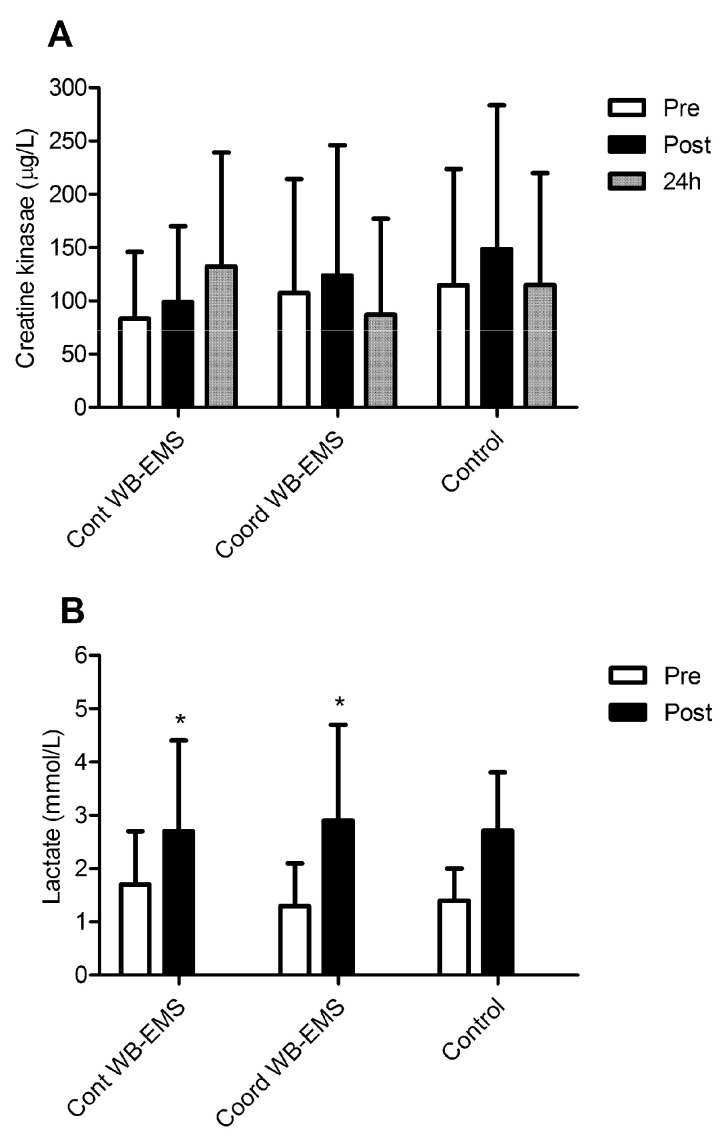
(**A**): creatine kinase blood levels before, after and the day after the strength exercise session with a continuous stimulus (Cont WB-EM), coordinated stimulus with concentric and eccentric phases (Coord WB-EMS) and without whole-body electromyostimulation (Control). (**B**): lactate blood levels before and after the strength exercise session with the continuous stimulus (Cont WB-EM), coordinated stimulus with concentric and eccentric phases (Coord WB-EMS) and without whole-body electromyostimulation (Control). * Significantly different than the pre-exercise in all trials (*p* < 0.05).

CK blood level (Figure 1A) results showed no significant differences between the trials. Furthermore, no differences were found at any time point within the main effects of the different experimental trials (F = 1.47, *p* = 0.243). The lactate blood levels (Figure 1B) significantly increased after exercise in the WB-EMS trials, but not in the Control, (F = 1.528, *p* = 0.040; post hoc: *p* = 0.042, *p* = 0.039 and *p* = 0.050 for the Cont WB-EMS, Coord WB-EMS and Control, respectively) with no significant differences among them (*p* = 0.281).

### 3.2. Heart Rate Variability

Regarding HRV data, the HRmean were significantly higher after the exercise in the three exercise trials, with no differences between trials. Additionally, we found that the PNN50 was significantly higher after the exercise in the Coord WB-EMS, compared to the Control. We found no other significant differences in the variables analyzed, between the groups before and after the exercise (Table 1).

### 3.3. Heart Rate, Blood Oxygen Saturation and the Rating of the Perceived Effort

**Figure 2:**
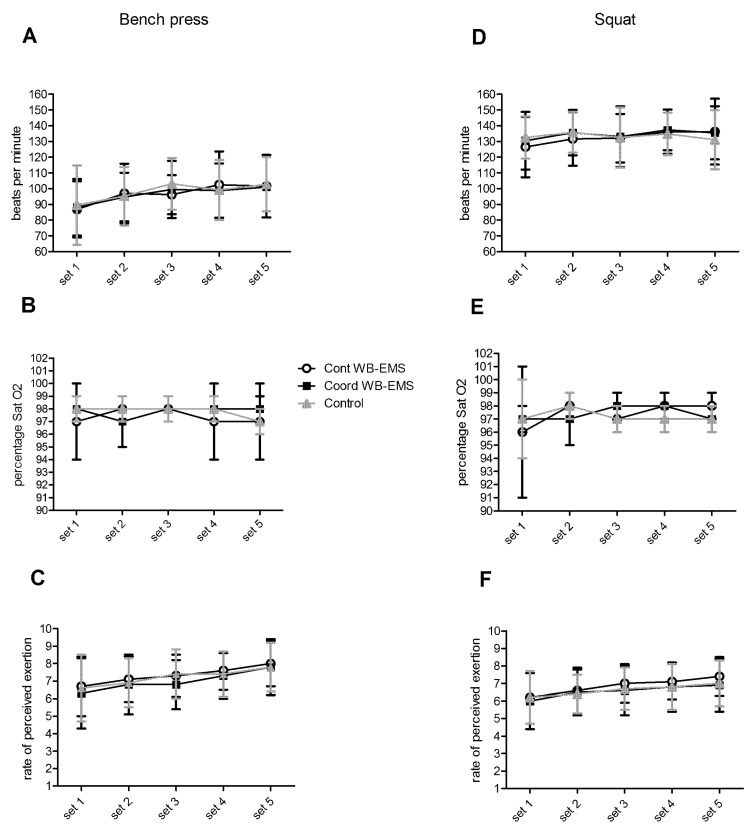
(**A**): heart rate, (**B**): percentage of the oxygen saturation, and (**C**): rate of the perceived exertion during the bench press exercise with the continuous stimulus (Cont WB-EM), coordinated stimulus with concentric and eccentric phases (Coord WB-EMS) and without whole-body electromyostimulation (Control). (**D**): heart rate, (**E**): percentage of the oxygen saturation, and (**F**): rate of the perceived exertion during the squat exercise with the continuous stimulus (Cont WB-EM), the coordinated stimulus with concentric and eccentric phases (Coord WB-EMS) and without whole-body electromyostimulation (Control).

During exercise, the HR did not significantly change in bench press exercise (F = 0.77, *p* = 0.452; Figure 2A) and in squat exercise (F = 1.02, *p* = 0.323; Figure 2D). Furthermore, that SatO2 remained similar between the trials, as well as during the bench press exercise (F = 5.07, *p* = 0.860; Figure 2B) and the squat exercise (F = 4.26, *p* = 0.380; Figure 2E). Finally, the RPE increased along with the sets, although not significantly and without differences between the trials in the bench press exercise (F = 0.77, *p* = 0.139; Figure 2C) and the squat exercise (F = 0.92, *p* = 0.222; Figure 2F).

### 3.4. Jump Tests, Isometric Handgrip Strength and Punching Speed

No significant differences were found in performance between trials (Table 2).

## 4. Discussion

The present crossover study was designed to analyze the effects of acute maximal strength training sessions with continuous (Cont WB-EMS) and coordinated with movement (Coord-WB-EMS) protocols of the whole body electromyostimulation with the same strength session without WB-EMS (Control) on muscle damage, fatigue and performance. The main finding of this study was that an acute strength training session with WB-EMS did not cause a greater elevation in the CK blood levels (i.e., muscle damage) than the same training session without WB-EMS, in the time period that it was monitored (i.e., 24 h). Furthermore, the use of the continuous (Cont WB-EMS) or coordinated (Coord-WB-EMS) stimulus did not make a difference in this fact. Moreover, the physiological variables assessed during the training sessions and the performance test results did not differ between the trials. Thus, this study supports that the WB-EMS training method, in healthy and physically active participants, does not disturb the usual physical and physiological dynamics of the maximal strength training. Our findings seem contrary to the extended idea of WB-EMS being harmful and dangerous for muscle health [8] in healthy and physically active participants.

To our knowledge, only one previous study performed a similar training and electrical impulse (85 Hz, 250/350 µs) protocols with healthy and physically active participants [30], however they used superimposed EMS instead of WB-EMS. In Wirtz et al.’s study, the participants performed 6-weeks of two sessions per week of four sets of squat exercises at a 10 RM intensity. They reported and compared the data from the first and the last sessions. The lactate blood levels in their first session were 8.0 ± 2.2 and 6.4 ± 2.3 mmol/L for the EMS and the control groups, respectively, and similar to us, with no significant difference between them. Bot, the current study using an exercise load of 90% 1RM (i.e., five repetitions) and the Wirtz et al.’s study using a 10 RM exercise load, demanded anaerobic metabolism. However, fatigue due to the phosphocreatine depletion was the likely factor that limited the repetitions for our participants, whereas the protocol of Wirtz et al., might have allowed more of a mix of the aerobic and anaerobic metabolisms that might have promoted a greater lactate production.

Regarding the CK blood levels, Wirtz et al. showed a similar increase after 24 h, in the EMS and control groups (+376 ± 471 and +204 ± 391 U/L, respectively). Again, our data showed lower levels than those in their study, but a similar behavior after 24 h. On the contrary, in another study on soccer players [7], WB-EMS was applied during the training sessions, consisting of 3 × 10 maximal squat jumps. The CK levels were measured pre, post and 24 h after the training, once a week for 14 weeks. The authors reported a significant increase in the CK levels after the WB-EMS training in the first session and in week 7, compared to the control group, reaching values of between 1000 and 2000 U/L. In any case, the CK blood levels reported in this study were within the common training breakpoint of 300–500 U/L [13] and far from the dangerous threshold of 5000 U/L, which indicates a risk of kidney harm [21]. Of note, a recent review about the exercise-induced muscle damage associated with the use of WB-EMS, has stated that the peak of the CK levels can occur after 72–96 h [31]. Thus, as we set our CK measurements post-exercise and 24 h later, it is possible that we could have missed greater values of the CK blood levels and thus, greater muscle damage.

The use of HRV to analyze the training intensity and to control the training status is widespread and is used in many sports [32,33,34,35]. In this line, the increase in the sympathetic autonomous tone would indicate the level of the training session intensity conducted. Focusing on the data obtained, we found that independent of the training used (continuous, coordinated and no WB-EMS) the autonomic modulation after the training sessions was not affected. This result highlighted how WB-EMS has a similar impact in the autonomic stress response than the lack of WB-EMS. The implementation of WB-EMS to trained participants in normal strength training, seems not to be as traumatic as the previous case studies showed, since the autonomic and biochemical responses of the athletes with and without WB-EMS were similar. Moreover, when comparing with the effect of other physical activities, such as high-intensity interval training, endurance probes, or other strength training on the HRV, we found that the use of WB-EMS presented a lower sympathetic activation [33,34,35]. It is shown how the habituation to the different training stimulus of the trained participants analyzed, allow them to have a normal psychophysiological response when the WB-EMS stimulus is applied, further when the acute resistance exercise volume is lower than 30 min [36].

To our knowledge, this is the first study analyzing the HR and BOS during WB-EMS training. Due to the impossibility of using heart rate monitors with the electromyostimulation suits, as these would be damaged and the data would be recorded incorrectly, it is difficult to monitor the HR during exercise. We used a pulse oximeter to measure the HR and BOS right after the end of each exercise set, however we recognize that this method could report values of the HR already decreasing because of the resting state. As we performed the same protocol on each of the three experimental trials, we could assume the same error for all of them. Nonetheless, the HR and BOS data were similar in the three trials.

The rate of perceived exertion is commonly used to control the intensity during the WB-EMS training [7,16,18,20,37]. We reported similar RPE data in the three experimental trials, slightly increasing along the sets in the bench press and the squat exercise, although with no significant differences. This is contrary to Kemmler et al. [16], who found that, in recreationally active young men, the use of WB-EMS during resistance training, incited a significantly higher RPE than the same session without WB-EMS (14.7 ± 1.5 and 11.9 ± 1.8, respectively). This difference between Kemmler’s study and ours may be due to the differences in the training protocol and the participants’ characteristics. In Kemmler’s study, they performed five dynamic exercises for large muscle groups, typically performed during commercial WB-EMS sessions, without any additional weights and structured in two sets of eight repetitions, with a total session time of 16 min. In the present study, we designed two exercises with five sets of five repetitions, but with a sufficient resting time between the sets to allow for the recovery. Regarding the participants’ characteristics, both studies recruited physically active people, but ours had a larger experience in strength training with high loads, although they were not strength athletes. 

Studies in elderly women, some at risk of sarcopenia, also reported higher RPE values, when using WB-EMS, compared to the control groups [14,15,17,37]. Nonetheless, this population is predisposed to show greater fatigue with increments in intensity as it is the addition of EMS to the training stimuli.

Finally, we measured some performance tests pre- and post-study, in order to analyze a possible performance impairment effect, due to WB-EMS. As we reported (Table 2) there were no differences in any test. Probably, the training load of the sessions was not large enough to induce a fatigue level to reduce performance in the chosen tests. Furthermore, as the exercise was set with a work-rest-ratio of 1:1, the WB-EMS units of the trials lasted about 10 min, while a regular session usually lasts for 20-min [31]. Thus, this undercurrent could also explain the absence of fatigue in the performance test. Nevertheless, the data reported in the present study show that the use of WB-EMS technology in strength training is without damage when the training protocol is accurately designed and supervised, and the users are healthy, physically trained participants. Conversely, the delivery of the electrical stimuli, continuous or coordinated with the muscle contractions, did not offer any advantage during training. Despite this, in order to consider WB-EMS as a proper technology for resistance training, future studies need to be carried out, mainly on the effects of the mid and long-term WB-EMS training programs on physical and sport performances. To end, this study is not free of limitations. Our measurement of the muscle damage reached 24 h post-exercise, while the peak of the CK levels could occur after 72–96 h. In addition, a recent study has shown that at least three adaptation sessions need to be performed before WB-EMS training in order to reach the maximum intensity tolerance [21]. Our design only included one familiarization session. Thus, added to the three trials of the experimental design, it is possible that our participants experienced some adaptations during the study that affected the results.

## 5. Conclusions

WB-EMS is relatively new training technology that has shown benefits mainly for health purposes, and less with performance targets. However, it has caused controversy, regarding the appropriateness of its use because it could be dangerous for muscle health. Consequently, coaches and exercise professionals need to assess the possible danger of its use when applying it to strength training at high loads. Defining the stimulus application protocol and adapting it to the exercise execution, as well as adapting the training load to the target population, is important to minimize this risk. According to the data report in this study, WB-EMS is a secure technology that can be implemented in strength training, since it did not produce greater muscle damage or physiological impairments than regular training. However, the longitudinal studies need to evaluate whether WB-EMS induces greater adaptations than regular strength training. Therefore, WB-EMS is a suitable technology that could be considered by exercise professionals as a tool to enhance muscle performance. Nevertheless, there are some recommended contraindications for the use of WB-EMS [22], such as pregnancy or recent surgery, that must be considered.

## Figures and Tables

**Table 1:** Changes (Mean ± SD) in the autonomic stress response after the strength exercise session with the continuous stimulus (Cont WB-EM), coordinated stimulus with concentric and eccentric phases (Coord WB-EMS) and without whole-body electromyostimulation (Control).

	Cont WB-EM			Coord WB-EMS			Control			Trial Main Effect			Time Main Effect			Interaction Effect		
	Pre Exercise	Post Exercise	% Change	Pre Exercise	Post Exercise	% Change	Pre Exercise	Post Exercise	% Change	F Value	*p* Value	η*p*^2^ Value	F Value	*p* Value	η*p*^2^ Value	F Value	*p* Value	η*p*^2^ Value
HRmean (bpm)	61.3 ± 5.5	71.7 ± 12.4 a	14.50	62.5 ± 15.7	72.7 ± 5.9 a	14.03	60.87 ± 9.9	73.4 ± 6.2 a	17.07	0.90	0.415	0.09	20.83	0.001	0.69	0.40	0.613	0.04
HRmin (bpm)	49.9 ± 4.6	51.0 ± 7.2	2.15	51.7 ± 9.2	50.7 ± 5.8	−1.93	50.9 ± 7.3	54.5 ± 8.5	6.60	1.30	0.296	0.12	1.35	0.275	0.13	0.47	0.614	0.05
HRmax (bpm)	91.9 ± 16.9	91.7 ± 16.1	−0.21	95.1 ± 20.5	94.5 ± 18.2	−0.63	91.5 ± 23.4	91.8 ± 17.0	0.32	0.07	0.919	0.01	3.38	0.099	0.27	0.26	0.757	0.03
RMSSD (ms)	85.7 ± 42.3	88.4 ± 37.4	3.50	81.5 ± 69.6	78.4 ± 36.8	−3.80	80.3 ± 45.4	68.2 ± 47.4	−15.06	0.68	0.472	0.07	0.51	0.493	0.05	0.43	0.573	0.04
PNN50 (%)	35.2 ± 15.6	37.9 ± 17.7	7.12	38.8 ± 24.7	41.6 ± 18.5 b	6.73	37.6 ± 17.5	35.1 ± 16.9	−6.64	1.46	0.626	0.05	2.25	0.168	0.20	12.09	0.020	0.90
LF (n.u.)	38.8 ± 14.9	38.2 ± 10.4	−1.54	38.1 ± 16.9	38.6 ± 14.2	1.29	42.4 ± 18.4	35.1 ± 13.2	−17.21	0.21	0.769	0.02	2.56	0.144	0.22	0.68	0.515	0.07
HF (n.u.)	55.7 ± 14.2	54.4 ± 11.0	−2.33	60.3 ± 17.4	60.3 ± 14.2	0.00	53.5 ± 17.0	63.1 ± 13.2	15.07	1.95	0.171	0.18	2.80	0.129	0.23	0.02	0.978	0.01
LF/HF (n.u.)	1.1 ± 0.8	1.1 ± 0.6	0.01	1.4 ± 1.2	1.2 ± 0.8	−14.28	1.6 ± 1.3	1.1 ± 0.7	−31.25	0.20	0.760	0.02	0.19	0.670	0.02	1.09	0.338	0.11
SD1 (ms)	42.1 ± 19.1	42.2 ± 17.6	0.23	34.9 ± 13.4	40.1 ± 17.1	12.69	39.5 ± 10.1	38.0 ± 13.7	−3.79	0.19	0.803	0.02	1.06	0.330	0.10	1.08	0.358	0.10
SD2 (ms)	63.4 ± 12.8	64.7 ± 14.2	2.00	58.5 ± 19.6	69.9 ± 10.7	16.30	67.3 ± 8.3	68.0 ± 16.2	1.02	1.76	0.205	0.16	0.89	0.370	0.09	0.06	0.939	0.01
ApEn	0.9 ± 0.3	1.0 ± 0.3	10.00	1.1 ± 0.3	0.9 ± 0.3	−10.00	1.1 ± 0.3	1.1 ± 0.3	0.00	0.30	0.697	0.03	0.65	0.439	0.07	1.16	0.331	0.11
SampEn	1.6 ± 0.3	1.3 ± 0.6	−18.76	1.5 ± 0.5	1.4 ± 0.4	−6.66	1.6 ± 0.4	1.4 ± 0.5	−12.5	0.55	0.535	0.06	0.96	0.352	0.09	0.75	0.476	0.07

HRmean: mean heart rate; HRmin: minimum heart rate; HRmax: maximum heart rate; RMSSD: square root of the average of the sum of the squared differences of the RR intervals; PNN50: percentage of the consecutive RR intervals that differ >50 ms; LF: low-frequency wave; HF: high-frequency wave; LF/HF: ratio between the low and high-frequency waves; SD1: variability of the short-term HRV; SD2: variability of the long-term HRV; ApEn: Approximate entropy; SampEn: Sample entropy. a: significantly different than the pre-exercise at the same exercise trial (all *p* = 0.001); b: significantly different than the Control group at the same moment (*p* = 0.019).

**Table 2:** Changes (Mean ± SD) in the squat jump (SJ), countermovement jump (CMJ) and Abalakov jump (ABK) tests, isometric handgrip strength (IHS), and punching speed (PS) after the strength exercise session with the continuous stimulus (Cont WB-EM), coordinated stimulus with concentric and eccentric phases (Coord WB-EMS) and without whole-body electromyostimulation (Control).

	Cont WB-EM			Coord WB-EMS			Control			Trial Main Effect			Time Main Effect			Interaction Effect		
	Pre Exercise	Post Exercise	% Change	Pre Exercise	Post Exercise	% Change	Pre Exercise	Post Exercise	% Change	F Value	*p* Value	η*p*^2^ Value	F Value	*p* Value	η*p*^2^ Value	F Value	*p* Value	η*p*^2^ Value
SJ (cm)	36.4 ± 6.7	37.7 ± 6.1	3.50	37.0 ± 6.5	37.0 ± 6.5	0.00	36.3 ± 6.9	37.1 ± 5.8	2.15	0.18	0.769	0.01	2.84	0.108	0.13	0.78	0.467	0.05
CMJ (cm)	38.9 ± 7.1	40.1 ± 6.8	2.99	39.0 ± 7.0	39.6 ± 7.4	1.51	38.6 ± 7.0	39.4 ± 6.4	2.03	1.08	0.347	0.05	0.74	0.398	0.04	0.22	0.803	0.05
ABK (cm)	42.7 ± 9.8	44.3 ± 9.6	3.61	43.2 ± 8.5	45.1 ± 9.6	4.21	42.9 ± 8.3	44.0 ± 8.8	2.50	0.88	0.422	0.04	0.54	0.470	0.03	0.97	0.386	0.05
IHS (Kg)	39.3 ± 11.0	39.6 ± 10.7	0.75	40.0 ± 11.2	40.3 ± 11.0	0.74	39.4 ± 10.4	40.3 ± 10.6	2.23	0.71	0.452	0.04	6.23	0.062	0.24	1.36	0.269	0.07
PS (number)	30.6 ± 7.8	31.1 ± 6.3	1.60	32.6 ± 7.0	30.7 ± 6.2	5.82	31.8 ± 8.0	31.9 ± 9.5	0.31	0.65	0.504	0.03	0.43	0.517	0.02	2.97	0.082	0.13

## Data Availability

All of the data are presented in the study.
